# Regenerative Therapien in der Rehabilitation mit den Schnittstellen Arbeitsleben und Geriatrie am Beispiel der extrakorporalen Stoßwellentherapie

**DOI:** 10.1007/s00132-026-04840-x

**Published:** 2026-05-12

**Authors:** Richard Crevenna

**Affiliations:** 1 Universitätsklinik für Physikalische Medizin, Rehabilitation und Arbeitsmedizin, Medizinische Universität Wien, Universitätsklinikum AKH Wien, Wien, Österreich; 2 Comprehensive Centers for Musculoskeletal Disorders, Medizinische Universität Wien, Universitätsklinikum AKH Wien, Wien, Österreich; 3 Kompetenzzentrum für Arbeitssicherheit und Gesundheitserhaltung, Universitätsklinikum AKH Wien, Wien, Österreich

**Keywords:** Chronischer Schmerz, Rehabilitation, Arbeitsfähigkeit, Geriatrie, Pflege, Chronic pain, Rehabilitation, Ability to work, Geriatrics, Nursing care

## Abstract

**Hintergrund:**

Die ESWT ist ein sicheres, evidenzbasiertes und effektives Verfahren, das sich in nahezu allen Bereichen der medizinischen Versorgung bewährt. Für einen qualitätsvollen Einsatz dieser effektiven Methode ist die Applikation entsprechend den geltenden Standards unerlässlich.

**Methodik:**

Traditionelle, narrative Übersicht.

**Ergebnisse:**

Der steigende Anteil körperlich belastender Berufe, zunehmende Bewegungsarmut, (Über‑)Alterung der Bevölkerung und Arbeitsverdichtung führen zu einer zunehmenden Zahl an Tendinopathien, myofaszialen Dysfunktionen und chronischen Schmerzzuständen. In diesem Kontext wird die ESWT zu einem relevanten Instrument einer modernen, funktionsorientierten Arbeits‑, Präventions‑, Rehabilitations- und Altersmedizin. ESWT wirkt zumeist als „Enabler“ und „Beschleuniger“, d. h. biologischer Aktivator funktioneller Prozesse. Die ESWT ist dabei kein passives Verfahren, sondern ein Aktivator, der durch Schmerzreduktion und regenerative Effekte funktionelle Behandlungen häufig erst ermöglicht. ESWT verbindet Regeneration, Prävention, Rehabilitation, Mobilität, Teilhabe und ökonomische Nachhaltigkeit und wird in einer alternden Gesellschaft weiter an Bedeutung gewinnen.

**Schlussfolgerungen:**

Die ESWT kann in der multimodalen Anwendung in Prävention, Rehabilitation, Arbeitsmedizin, Geriatrie und Pflege hohe Relevanz haben. Eine vermehrte und durch das öffentliche Gesundheitssystem refundierte operative Nutzung in der Präventions‑, Reha- und Arbeitsmedizin sowie der Geriatrie sind anzustreben.

## Abstract (en)

**Background:**

ESWT is a safe, evidence-based, and effective procedure that has proven itself in almost all areas of medical care. To ensure high-quality use of this effective method, it is essential that it is applied in accordance with the applicable standards.

**Methodology:**

Traditional, narrative review.

**Results:**

The increasing proportion of physically demanding occupations, increasing lack of exercise, (over)aging of the population, and work intensification are leading to an increasing number of tendinopathies, myofascial dysfunctions, and chronic pain conditions. In this context, ESWT is becoming a relevant tool in modern, function-oriented occupational, preventive, rehabilitative, and geriatric medicine. ESWT usually acts as an “enabler” and “accelerator”, i.e., a biological activator of functional processes. ESWT is not a passive procedure, but rather an activator that often enables functional treatments through pain reduction and regenerative effects. ESWT combines regeneration, prevention, rehabilitation, mobility, participation, and economic sustainability and will continue to gain importance in an aging society.

**Conclusions:**

ESWT can be highly relevant in multimodal applications in prevention, rehabilitation, occupational medicine, geriatrics, and nursing care. Increased use of ESWT in prevention, rehabilitation, occupational medicine, and geriatrics, with reimbursement by the public health system, should be encouraged.

Die sog. Extrakorporale Stoßwellentherapie (ESWT) ist ein Verfahren, für das in den letzten Jahren immer mehr Anwendungsmöglichkeiten gefunden wurden. Die ESWT wird in der Prävention, Rehabilitation, Arbeitsmedizin, Geriatrie und Pflege bei vielfältigen Problemen und Beschwerden eingesetzt. Sie ist ein nichtinvasives, risikoarmes, gut steuerbares Verfahren, für dessen Wirksamkeit immer mehr Evidenz vorliegt.

## Einführung

Die „extrakorporale Stoßwellentherapie (ESWT)“, Schlüsseltechnologie der regenerativen physikalischen Mechanotherapie hat sich seit den 1980er-Jahren vom urologischen Verfahren zur Lithotripsie zu einer der fundiertesten physikalischen Technologien in der Medizin entwickelt. Sie ist ein nichtinvasives, risikoarmes, gut steuerbares und biologisch nachvollziehbares Verfahren, dessen Wirksamkeit sich mit modernen Erkenntnissen der Zellbiologie und Mechanotransduktion deckt. Dies macht sie zum idealen Partner für multimodale Therapiestrategien in Prävention, Rehabilitation und Public Health. Heute ist sie eine zentrale Säule der Mechanotherapie oder -medizin, also jener Disziplin, die mechanische Stimuli nutzt, um biologische Regenerationsprozesse in muskulären, tendinösen, faszialen und knöchernen Strukturen zu modulieren. Fachgesellschaften wie die DIGEST und ISMST haben klare, evidenzbasierte Standards für Indikationen, Kontraindikationen und die optimale Anwendung etabliert [1–14].

## Mögliche Relevanz der ESWT in Feldern wie Arbeitsmedizin, Geriatrie und Pflege

Die wachsende klinische Evidenz in Orthopädie und Traumatologie, Rehabilitation, Dermatologie (u. a. Wundheilung) sowie ausgewählten neurologischen und mittlerweile auch onkologischen Bereichen unterstützt die zunehmende Bedeutung der ESWT. Die ESWT bietet mögliche Antworten auf die zentralen Herausforderungen moderner Gesundheitssysteme (Tab. 1) und hat ihren Platz in der multimodalen Anwendung in der Prävention, Rehabilitation, Arbeitsmedizin, Geriatrie, Pflege sowie höchst relevante sozialmedizinische und ökonomische Effekte.

**Tab. 1:** Zentrale Herausforderungen moderner Gesundheitssysteme

Demografischer Wandel mit Überalterung
Steigende Prävalenz muskuloskelettaler Erkrankungen
Zunahme chronischer Schmerzen
Zunehmender Rehabilitationsbedarf nach Operationen, Unfällen und Tumortherapie
Steigende Arbeitsbelastung in vielen Berufsgruppen
Fachkräftemangel in Pflege und Medizin
Wachsende wirtschaftliche Belastung der Gesundheits- und Sozialversicherungssysteme
Eingeschränkte und schwindende Ressourcen in allen Bereichen

Mechanotransduktion als biologischer Kernmechanismus bedeutet die Umwandlung eines mechanischen Stimulus in eine zelluläre Antwort. Die ESWT beeinflusst eine Vielzahl mechanosensitiver Strukturen wie Integrine, Piezo1-/Piezo2-Kanäle, TRPV4-Kanäle, zytoskelettale Aktin-Netzwerke und die Kernmembranmechanik etc. Dies führt u. a. zur Aktivierung zentraler Biomoleküle wie YAP/TAZ, MAPK/ERK, PI3K/Akt, HIF-1α, eNOS, VEGF, was zu biologischen Effekten wie einer verbesserte Mikrozirkulation, Angiogenese, Zellproliferation, Modulation chronischer Entzündungsprozesse, Differenzierung gewebstypischer Zellen, Rekrutierung mesenchymaler Stammzellen und zu einem verbesserten extrazellulären Matrix(ECM)-Remodelling führen und strukturbiologische Reparaturprozesse initiieren kann. Analgetische Effekte, d. h. Schmerzlinderung erfolgt u. a. durch Reduktion von Substanz P, Modulation nozizeptiver Fasern, myofasziale Relaxation, Senkung lokaler Entzündungsprozesse und segmentale inhibitorische Effekte. Die Schmerzreduktion ermöglicht eine frühzeitige funktionelle Therapie und kann somit als Enabler einen wesentlichen Rehabilitationsmotor darstellen.

Standardindikationen der ESWT umfassen u. a. Plantarfasziitis, Achillodynie, Epicondylitis radialis, Kalkschulter, Patellaspitzensyndrom, „greater trochanteric pain syndrome“, verzögerte Frakturheilung, Pseudoarthrosen und chronische Wunden. Weitere relevante Indikationen stellen die Rotatorenmanschetten-Tendinopathie ohne Kalzifikation, das Peronealsehnensyndrom, Schienbeinkantensyndrom und myofasziale Syndrome sowie sog. Expert:innenindikationen wie früharthrotische Beschwerden, spastische Syndrome (adjuvant), Polyneuropathie, Lymphödem und Dupuytren‑/Ledderhose-Frühstadien dar [1–14].

## Anwendung der ESWT im präventionsmedizinischen Gesamtkontext [2, 3, 15–17]

Die Prävention muskuloskelettaler Erkrankungen gewinnt im 21. Jahrhundert an enormer Bedeutung. Der steigende Anteil körperlich belastender Berufe, zunehmende Bewegungsarmut, (Über‑)Alterung der Bevölkerung und Arbeitsverdichtung führen zu einer zunehmenden Zahl an Tendinopathien, myofaszialen Dysfunktionen und chronischen Schmerzzuständen. In diesem Kontext wird die ESWT zu einem relevanten Instrument einer modernen, funktionsorientierten Präventionsmedizin. Wichtig ist dabei, dass die ESWT keine Bewegung, kein Training und keine Arbeitsplatzadaptation ersetzt – sie erleichtert und beschleunigt diese aber, indem sie biologische Voraussetzungen für Belastbarkeit verbessert.

Unter Primärprävention, d. h. die Funktionssicherung vor struktureller Schädigung versteht man das Verhindern des Auftretens einer Erkrankung bei noch bis dato noch nicht Betroffenen. ESWT gilt dabei zwar nicht als allgemeine, primärpräventive Vorsorgemaßnahme für Gesunde, aber als ein wirksames präventives Tool für Risikogruppen. Primärprävention bedeutet dabei die gezielte Anwendung bei Personengruppen mit hoher funktioneller Belastung, wie Pflegekräften, Baugewerbe, Handwerk, Leistungssport, Menschen ab 50 mit degenerativer Frühdynamik etc., mit den Zielen einer Stabilisierung der Strukturen, Verbesserung der Perfusion und Erhalt bzw. Verbesserung der Belastbarkeit. Die ESWT spielt demnach zwar keine Rolle als allgemeine Prophylaxe bei gesunden Menschen, sehr wohl aber als gezielte präventive Maßnahme bei Risikogruppen, bei denen bereits erste funktionelle, noch reversible Veränderungen erkennbar sind. Zu diesen Risikogruppen zählen „beruflich Hochbelastete“ wie Pflegekräfte (ständiges Heben, Transferieren etc.), Handwerk und Baugewerbe (Überkopf‑, Knie- und Achillessehnenbelastung), Landwirtschaft (vibrierende Maschinen, monotone Bewegungen), Logistik und Transport (Dauerbelastungen, Zwangshaltungen), sportlich Hochbelastete wie Ausdauersportler, Sprung‑, Lauf- und Kraftsportler (Achillessehnen, Schulter, Rücken) sowie Racket-Sportarten (Schulter, Ellenbogen) und Altersgruppen mit erhöhtem Degenerationsrisiko wie Personen über 50 Jahre (Tendinopathierisiko steigt hier deutlich) und Patient:innen mit metabolischen Risikofaktoren (Diabetes, Adipositas). Die ESWT unterstützt hier durch eine Verbesserung der lokalen Mikrozirkulation, Auflösung subklinischer Mikroentzündungen, Förderung der mechanischen Belastbarkeit, Stabilisierung der extrazellulären Matrix mit Mobilisation zu aktivem Training und wirkt dabei additiv fördernd – nicht ersetzend – also ideal im Zusammenspiel mit Training, Mobilisation und ergonomischen Maßnahmen.

In der Sekundärprävention kann die ESWT helfen, die Chronifizierung von Erkrankungen verhindern

In der Sekundärprävention als klinisch relevantester Ebene kann die ESWT die Chronifizierung von Erkrankungen, Schmerzkarrieren, Funktionsverlust und Arbeitsunfähigkeit mit Arbeitsausfall durch Anwendung bei ersten Tendinopathiezeichen, überlastungsinduzierten Schmerzen und myofaszialen Verspannungsmustern verhindern helfen. Dies ist der wichtigste präventive Anwendungsbereich und als typische Situationen gelten erste belastungsabhängige Schmerzen, sporadische Achillessehnenbeschwerden, eine beginnende Plantarfasziitis, leichte myofasziale Schulter- oder Nackenbeschwerden oder funktionelle Einschränkungen ohne strukturelle Schäden. ESWT ist hier so effektiv, weil sie die Entwicklung eines „degenerativen Circulus vitiosus“ unterbricht, lokale Entzündungsprozesse frühzeitig reduziert, die Sehnen- und Bindegewebsversorgung verbessert und funktionelles Training zu einem Zeitpunkt, an dem Training besonders wirksam ist, ermöglicht („Enabler“-Funktion). Gelingt diese Phase gut, kann ein Großteil der Tendinopathien ohne Chronifizierung abgefangen werden – dies bedeutet eine enorme medizinische und ökonomische Entlastung.

Die Tertiärprävention, als Stabilisierung nach erfolgter Therapie erfolgt nach abgeschlossener Therapie z. B. als Booster-ESWT zur Funktionssicherung und zur Reduktion von Rezidiven, stellt einen Enabler in der Rehabilitation dar und ist besonders wertvoll zur Verbesserung der Arbeitsfähigkeit mit Return-to-Work (RTW) sowie zur Verbesserung der sozialen Teilhabe. Nach erfolgreicher Therapie ist es entscheidend, die funktionsbiologische Stabilität langfristig zu sichern. Die ESWT bietet hier u. a. strukturelle Festigung nach Sportverletzungen, Stabilisierung degenerativer Sehnenveränderungen, Unterstützung von Reha-Übergängen (von stationär zu ambulant) und eine Reduktion von Rezidiven, präventive Stabilisierung bei wiederkehrender Achillodynie oder eine ergänzende Behandlung myofaszialer Dysfunktionen in Belastungsperioden.

Quartärprävention wird im Kontext moderner Gesundheitssysteme immer zentraler und bedeutet die Vermeidung von Überbehandlung und Überversorgung, d. h. von nicht notwendigen medizinischen Maßnahmen, wie u. a. Vermeidung unnötiger Eingriffe, d. h. Operationen, wiederholten Steroidinfiltrationen, Schmerzmitteldauertherapie, übermäßige Bildgebung ohne therapeutischen Mehrwert etc. Ein solches „overdoctoring“ kann durch ESWT effektiv verhindert werden. Dies schützt besonders multimorbide, ältere oder onkologische Patient:innen, da damit operative Risiken und medikamentöse Nebenwirkungen vermieden werden können, aber durch Reduktion o. g. Kostentreiber gleichzeitig auch unser Gesundheits- und Sozialsystem, indem Überversorgung vermieden werden kann.

## ESWT in der Rehabilitation [2, 3, 18–26]

In der Rehabilitation wirkt ESWT als „Enabler“ und „biologischer Beschleuniger“, d. h. biologischer Aktivator funktioneller Prozesse. Die ESWT ist in der Rehabilitation kein passives Verfahren, sondern ein Regenerationsaktivator, der erst funktionelle Behandlungen ermöglicht. Die Rehabilitation umfasst u. a. orthopädische, traumatologische, neurologische und onkologische Felder – jeweils mit spezifischen Ansätzen.

In der orthopädisch-traumatologischen Rehabilitation ermöglicht die ESWT eine raschere Schmerzlinderung, erleichterte Mobilisation, Beschleunigung struktureller Heilung sowie Unterstützung der Rehabilitation. Hier wirkt die ESWT auf drei Ebenen gleichzeitig: auf jener der Schmerzlinderung (bessere Mitarbeit), jener der Strukturstimulation (beschleunigte Heilung) und jener der Funktionsverbesserung (frühere Belastbarkeit). Indikationen stellen u. a. Tendinopathien, Muskelverletzungen, postoperative Beschwerden (z. B. nach Rotatorenmanschettennaht), Patellaspitzensyndrom, Achillodynie und verzögerte Frakturheilung („non-unions“, Pseudoarthrosen) dar. Die ESWT erleichtert es Patient:innen, frühzeitig aktiv zu trainieren, funktionelle Muster zu normalisieren und in den Alltag und Beruf zurückzukehren. Sie wirkt als Enabler bzw. Katalysator für die gesamte Rehabilitationskette.

In der neurologischen Rehabilitation kann die ESWT einen additiven Einsatz u. a. bei spastischen Syndromen, Mobilisationsproblemen, myofaszialen Beschwerden, funktionellen Asymmetrien und neurodegenerativen Erkrankungen finden, wobei die ESWT die neurologische Therapie effektiv unterstützen kann, d. h. es geht primär um adjuvante Effekte, wie Reduktion spastikassoziierter Muskelverhärtungen, Verbesserung der Mobilisation, Erleichterung pflegerischer Tätigkeiten und Förderung von Gangbild und Bewegungsmustern. Die ESWT ersetzt keine neurologische Therapie, kann aber die biomechanischen Voraussetzungen verändern.

In der onkologischen Rehabilitation ist ein aktiver maligner Tumor im Fokus zwar eine Kontraindikation der ESWT – dies ist sowohl in der DIGEST- als auch in der ISMST-Leitlinie klar definiert – gleichzeitig profitieren gerade onkologische Patient:innen erheblich von der ESWT, wenn diese zur Behandlung funktioneller, muskulärer, faszialer oder tendinöser Einschränkungen und Defizite eingesetzt wird. Sinnvoll ist die ESWT zur Behandlung muskuloskelettaler Folgeprobleme sowie von Folgen der Tumorerkrankung bzw. der überlebensnotwendigen Krebstherapie wie Operationen, Strahlentherapie, systemische Therapien (Immobilität, myofasziale Verkürzungen, postoperative Narben, Lymphödem, sensomotorische Dysfunktionen wie Polyneuropathie). In der onkologischen Rehabilitation stehen häufig funktionelle muskuläre und fasziale Folgestörungen im Vordergrund, wobei es durch Operationen, Bestrahlung oder Chemotherapie zu Bewegungseinschränkungen, myofaszialen Verspannungen, Narbenadhäsionen, neuropathischen Begleitsymptomen und sekundären Tendinopathien kommen kann. Als typische Lokalisation innerhalb der onkologischen Rehabilitation gilt die Schulterregion nach Brustkrebstherapie. Postmastektomie-Syndrome führen oft zu myofaszialen Dysbalancen, Kompensationsmustern und sekundären Tendinopathien. Die ESWT kann hier durch Reduktion myofaszialer Triggerpunkte, Verbesserung der Mobilität und Unterstützung bei aktiven Übungen die Rehabilitation erleichtern. Am Beckengürtel wiederum, z. B. nach urologischen bzw. gynäkologischen Tumoren findet die ESWT ihren Einsatz nach radikaler Prostatektomie und nach Zervixkarzinom- oder Ovarialkarzinomoperationen, wo häufig myofasziale Verspannungen, lumbopelvine Dysfunktionen und sekundäre Hüft- oder Adduktoren-Tendinopathien entstehen. Eine ESWT ermöglicht hier eine myofasziale Entspannung, funktionelle Korrektur und wirkt als „Enabler“ von Gangschulung und Training.

Nach Strahlentherapie kann es zu strahlentherapiebedingten Weichteilveränderungen wie Lymphödemen, Fibrosen und Bindegewebsverhärtungen als Strahlenspätreaktionen kommen. Hier kann die ESWT zur Verbesserung der Mikrozirkulation, Förderung der dynamischen Neugestaltung des ECM-Remodellings und zum Lösen fibröser Strukturen eingesetzt werden. Bei onkologischer Fatigue und Inaktivitätsfolgen wirkt ESWT indirekt durch die Schmerzreduktion (ermöglicht Aktivität) und als „Enabler“ des Trainings mit Unterstützung des Aufbaus funktioneller Leistungsfähigkeit, was besonders relevant ist, da dies eine der wichtigsten Maßnahmen gegen tumorbedingte Fatigue ist und zusätzlich zu einem verbesserten krebsspezifischen Überleben führen kann.

Die ESWT ist in der onkologischen Rehabilitation relevant, weil viele onkologische Patient:innen multimorbide sind. Gleichzeitig reagieren einige ablehnend auf zusätzliche medikamentöse und invasive Verfahren und benötigen daher sichere und nebenwirkungsarme Behandlungsalternativen. Die ESWT erfüllt diese Anforderungen, denn sie ist nichtinvasiv, pharmakologisch neutral, gut verträglich, hat keine systemischen Nebenwirkungen – und ermöglicht Bewegung und damit gebesserte Funktionsfähigkeit und Teilhabe. Die ESWT führt zu einem reduzierten Schmerzmittel- bzw. Opioidbedarf, ist ökonomisch effizient und gut integrierbar in komplexe Reha-Pfade.

## ESWT in der Arbeitsmedizin [2, 3, 27]

Die Arbeitsmedizin befasst sich mit der Erhaltung von Gesundheit, Leistungsfähigkeit und Teilhabe im beruflichen Kontext. Hier spielt die ESWT eine wachsende Rolle als Werkzeug der Funktionserhaltung und beruflichen Teilhabe. Typische Risikogruppen der Arbeitsmedizin stellen u. a. Pflegekräfte (v. a. Schulter, Lendenwirbelsäule), Industrie und Montage (v. a. Unterarm, Ellenbogen), Handwerk und Baugewerbe (v. a. Achillessehne, Knie), Büroarbeit (v. a. Nacken, Triggerpunkte) etc. dar. Es geht hier um das Verbleiben bzw. um die Rückkehr an den Arbeitsplatz (RTW) durch schnelle Schmerzreduktion, Funktionsverbesserung, Verringerung psychosozialer Belastungsfaktoren und geringere Chronifizierungsraten. Eine ESWT kann im Rahmen des sog. betrieblichen Eingliederungsmanagements (BEM) die funktionale Gesundheit durch eine Förderung der beruflichen Teilhabe durch positive Effekte auf Körperfunktionen (Strukturebene), Aktivitäten (Bewegung, Kraft, Belastbarkeit) und Teilhabe (Arbeitsfähigkeit, Berufs- und Sozialleben) im Sinne des biopsychosozialen Modells der modernen Arbeitsmedizin unterstützen.

Die Belastungsprofile relevanter Berufsgruppen unterscheiden sich und führen bei den Pflegeberufen durch repetitive Lasten, Überkopfarbeit, Heben und Bewegen von Patient:innen häufig zu Schulter-Arm-Tendinopathien und lumbalen myofaszialen Syndromen, im Baugewerbe und Handwerk durch Vibrationen, einseitige monotone Belastungen sowie Überkopfarbeiten u. a. zur Achillodynie, Rotatorenmanschettenproblemen, Epikondylopathien etc. In Industrie und Montage führen repetitive Tätigkeiten und lokale Fehl- und Überlastungen v. a. zu Unterarm- und Ellenbogentendinopathien und im Büro sowie bei PC-Tätigkeiten monotone Haltungen v. a. zu myofaszialen Nacken- und Schulterbeschwerden und zu myofaszialen Schmerzsyndromen. Die ESWT adressiert alle diese Diagnosen zielgerichtet, insbesondere in frühen Stadien und kann so eine Chronifizierung verhindern helfen.

Eine ESWT sollte einfach in BEM-Prozesse integriert werden können, da sie planbar, effizient, leitlinienkonform und kostenschonend ist. Sie verkürzt RTW-Zeiten durch rasche Schmerzsenkung (entscheidend für Arbeitsfähigkeit), Verbesserung der Belastbarkeit mit gebessertem Muskel-Sehnen-Verhalten bei Belastung und Auflösung myofaszialer Muster (oft zentral bei berufsbedingten Beschwerden), Integration in arbeitsplatzbezogene Rehabilitationsprozesse (z. B. arbeitsplatzspezifisches Training) und psychosoziale Stabilisierung (Schmerzen sind führender Prädiktor für Angstvermeidungsverhalten).

## ESWT in der alternden Gesellschaft, Geriatrie und Pflege [2, 3, 28–32]

In der Geriatrie sind Themen wie Mobilitätserhalt und Sturzprävention besonders relevant. Mobilität ist im Alter ein wichtiger gesundheitlicher Prädiktor für Sturzrisiko, Pflegeabhängigkeit, Lebensqualität und Mortalität. Eine ESWT reduziert Schmerzen und verbessert die Beweglichkeit bei Tendinopathien, degenerativen Beschwerden, myofaszialen Dysfunktionen und postoperativen Beschwerden und führt in der Sturzprävention zu Verbesserungen durch eine dadurch schmerzfreiere Mobilität, bessere Muskelaktivierung, Verbesserung der Gleichgewichtsreaktionen und Reduktion von Schonhaltungen. Weiters kann die ESWT den Bedarf an nichtsteroidale Antirheumatika, Opioiden, Kortikosteroidinjektionen etc. reduzieren und die Polypharmazie positiv beeinflussen. Im Bereich Pflege könnte die ESWT die Entlastung der Versorgungssysteme unterstützen, da ESWT zu leichterer Mobilisation, höherer Kooperationsfähigkeit, geringeren pflegerischen Belastungen, Reduktion immobilitätsbedingter Komplikationen (z. B. Dekubitus) und einer Verzögerung der Pflegebedürftigkeit führen und damit sowohl die professionelle Pflege als auch pflegende Angehörige entlasten kann.

Die Möglichkeiten der ESWT im pflegerischen Versorgungskontext sind vielfältig, aber noch unterschätzt

Die Alterung der Bevölkerung führt zu einer massiven Zunahme muskuloskelettaler Erkrankungen. Geriatrische Patient:innen profitieren besonders von der ESWT, weil diese Therapie wenig Nebenwirkungen verursacht, keine systemische Belastung darstellt, die Mobilität verbessert, die Selbstständigkeit fördert und die Pflegebedürftigkeit verzögern kann. Typische geriatrische Problemfelder stellen degenerative Tendinopathien (Schulter, Achillessehne, Glutealsehnen etc.), myofasziale Syndrome (altersbedingte Strukturveränderungen führen zu kompensatorischen Verspannungen), Gang- und Gleichgewichtsprobleme durch Schmerzen (Schmerz führt zu Inaktivität – das Sturzrisiko steigt) und Polypharmazie (ältere Menschen sind da besonders gefährdet) dar. In der Sturzprävention wirkt ESWT indirekt, indem sie schmerzfreie Gehstrecken verlängern und die Standstabilität, funktionelle Beweglichkeit und Hüft- und Beckenmuskelfunktion verbessern kann. Da Schmerz einer der stärksten Prädiktoren für Stürze ist, wirkt die ESWT hier mittelbar sturzpräventiv. In der Pflegeprävention wirkt ESWT durch Funktionsverbesserung, Schmerzreduktion mit erhöhter Mobilität und kann so zur Verzögerung oder Vermeidung von Pflegebedürftigkeit beitragen.

Die Möglichkeiten der ESWT im pflegerischen Versorgungskontext sind vielfältig, aber noch unterschätzt. Die Pflege ist ein zunehmend belasteter Sektor – die demografische Entwicklung, steigende Multimorbidität, wachsende Pflegefallzahlen und hoher körperlicher sowie psychischer Arbeitsdruck prägen den Berufsalltag. Pflegebedürftige Menschen wiederum leiden häufig unter Mobilitätseinschränkungen, Schmerzen, Funktionsverlust und immobilitätsbedingten Komplikationen. Die ESWT ist zwar kein originäres Pflegeverfahren, aber sie hat einen hohen indirekten Stellenwert für pflegerische Versorgungskonzepte, da sie die Mobilität verbessern, Schmerzen reduzieren, die funktionelle Selbstständigkeit stärken, den Pflegeaufwand senken und pflegerische Tätigkeiten erleichtern kann. Dies ist sowohl für die professionelle Pflege als auch für pflegende Angehörige hochrelevant. Die Reduktion des pflegeassoziierten Muskel-Skelett-Aufwands hat zwei Aspekte. Viele pflegebedürftige Personen haben degenerative Beschwerden, Tendinopathien, myofasziale Schmerzen, Funktionseinbußen nach Immobilität oder Krankenhausaufenthalten sowie Schmerzen in Schulter, Knie, Hüfte oder Achillessehne. ESWT kann diese Beschwerden strukturell und funktionell verbessern. Als praxisrelevanter Nutzen ableitbar sind u. a. weniger Schmerzen im Transfer, bessere Mitarbeit bei Mobilisation, längere Erhaltung der Selbstständigkeit und eine Unterstützung bei Prophylaxen (Dekubitus, Kontrakturen, Thrombosen durch höhere Aktivität). Professionelle Pflegekräfte sind eine der Berufsgruppen mit der höchsten Prävalenz muskuloskelettaler Erkrankungen. Zahlreiche Studien zeigen extrem hohe Raten von Schultersehnenproblemen, Rückenbeschwerden wie Kreuzschmerz, Nackenmyogelosen und Epikondylopathien. Durch eine ESWT können pflegende Personen mit frühen oder chronischen tendinösen Beschwerden schneller wieder belastbar werden, wodurch Krankenstände reduziert, die Dienstplanstabilität erhöht und Berufsausstiege verzögert werden können. Dies ist für die arbeitgebenden Einrichtungen ebenso relevant wie für die Betroffenen selbst. Die ESWT kann auch für die Pflege im häuslichen Umfeld relevant sein. Für pflegende Angehörige kann sie weniger körperliche Belastung beim unterstützten Transfer, bessere Gestaltungsfähigkeit des Pflegealltags und eine höhere Lebensqualität in langdauernden Pflegesituationen bedeuten. Insbesondere im häuslichen Umfeld, wo professionelle Strukturen fehlen, ist die Entlastung durch Verbesserung der Patient:innenfunktion von enormem Wert.

## Gesundheits- und sozioökonomische Bedeutung der ESWT

Die ökonomische Relevanz der ESWT hat in den letzten Jahren stark zugenommen. Sie ergibt sich aus einer Kombination struktureller Effekte wie eine Vermeidung invasiver Therapien, Reduktion chronischer Krankheitsverläufe, Verringerung von Arbeitsunfähigkeit, Stärkung der Teilhabe am Berufsleben, Verzögerung von Pflegebedürftigkeit und eine geringere Belastung der Gesundheits- und Sozialversicherungssysteme. Der volkswirtschaftliche Nutzen ergibt sich nicht primär aus der Einzelbehandlung, sondern aus der Systemwirkung, die sich aus der Funktionsverbesserung mit verbesserter Mobilität und Teilhabesteigerung ergibt. Direkte Kosteneffekte entstehen durch die Verringerung operativer Eingriffe. Viele Tendinopathien und degenerative Beschwerden erfordern nach ESWT keine Operation mehr, sofern sie leitlinienkonform angewendet wird – insbesondere gilt dies für Plantarfasziitis, Kalkschulter, Achillodynie, Patellaspitzensyndrom. Auch verhindert eine ESWT häufig die Notwendigkeit wiederholter Kortisoninjektionen (Nebenwirkungen u. a. Degeneration, Fettgewebsatrophie, Infektrisiken) und führt zu geringeren Kosten für Bildgebung. Chronische Tendinopathien führen oft zu wiederholten MRT-, CT- oder Ultraschalluntersuchungen. Eine strukturierte ESWT stabilisiert Verläufe und reduziert diese oft ausufernde Diagnostik. Auch geringere Arzneimittelkosten, ein reduzierter Bedarf an nichtsteroidalen Antirheumatika , Opioiden und Muskelrelaxantien ist gerade bei älteren Patient:innen relevant. Weitere direkte Kosteneffekte resultieren aus der Reduktion stationärer Aufenthalte insbesondere bei schweren Schmerzsyndromen, bei Wundheilungsstörungen und bei funktionellen postoperativen Problemen.

Indirekte Einsparungen sind volkswirtschaftlich besonders relevant und erfolgen durch eine Reduktion von Fehlzeiten (arbeitsmedizinisch bedeutsam – hier sind erhebliche Einsparungen möglich), Sicherung der Arbeitsfähigkeit (durch Verhinderung chronischer Verläufe und Reduktion von Frühpensionierungen), geringere Sturzraten im Alter (durch bessere Mobilität und weniger Frakturen, Operationen, Reha-Kosten), Hintanhaltung bzw. Verzögerung von Pflegebedürftigkeit und eine Verringerung sozialmedizinischer Folgekosten. Es resultieren weniger Berufsunfähigkeit und Arbeitsausfall durch Kurzzeit- und Langzeitkrankschreibungen sowie sozialrechtliche Ersatzleistungen. Über längere Zeiträume führt die ESWT zu gesamtwirtschaftlichen Systemeffekten wie einer Erhöhung gesunder Lebensjahre („healthy life years“), Stabilisierung der Pflegeversicherung, geringerer Belastung der Rentenversicherung und einer Entlastung des ambulanten und stationären Gesundheitssystems (Tab. 2).

**Tab. 2:** Gesundheitsökonomische Aspekte bei Einsatz der ESWT

Direkte Einsparungen	Reduktion operativer Eingriffe
	geringere Kosten für bildgebende Verfahren wie u. a. MRT, CT
	Reduktion des Medikamenteneinsatzes
	Reduktion der Injektionsfrequenz
	Reduktion von Operationen
Indirekte Einsparungen	Reduktion von Arbeitsunfähigkeit
	Reduktion von Chronifizierung
	Reduktion der Reha-Kosten
	Reduktion von Sturzfolgen
	Reduktion von Pflegeheimaufenthalten
Makroökonomische Effekte	Erhalt der Arbeitsfähigkeit
	Verlängerung der gesunden Lebensjahre
	Entlastung der Pflegeversicherung
	Senkung langfristiger Gesundheits- und Sozialkosten

## ESWT in multimodalen Therapieansätzen

Für die ESWT empfiehlt sich im multimodalen Setting die Kombination mit anderen physikalischen, regenerativen Verfahren wie u. a. Bewegungs- und sog. Medizinischer Trainingstherapie, Manueller Therapie, Modalitäten aus der Elektrotherapie, gepulsten Magnetfeldern wie Ioneninduktions- und Mechanotransduktionstherapie, Photobiomodulation sowie auch mit „platelet-rich plasma“ etc.

Zu den Zukunftsperspektiven der ESWT gehören u. a. KI-gestützte Dosierungsmodelle, individualisierte Mechanotransduktionsprofile (im Sinne gelebter personalisierter Präzisionsmedizin), Integration in die telemedizinische Rehabilitation, größere internationale, auch die Prävention, Rehabilitation sowie Arbeitsmedizin verbindende, Register (z. B. über DIGEST und ISMST) mit weiterer Standardisierung der Protokolle und eine entsprechend vermehrte, durch das öffentliche Gesundheitssystem refundierte operative Nutzung in der Präventions- und Arbeitsmedizin sowie der Geriatrie.

## Fazit für die Praxis

- Die extrakorporalen Stoßwellentherapie (ESWT) ist ein sicheres, evidenzbasiertes und effektives Verfahren, das sich in nahezu allen Bereichen der medizinischen Versorgung bewährt.
- Die ESWT verbindet Regeneration, Prävention, Rehabilitation, Mobilität, Teilhabe und ökonomische Nachhaltigkeit und wird in einer alternden Gesellschaft weiter an Bedeutung gewinnen.
- Für einen qualitätsvollen Einsatz dieser effektiven Methode ist die Applikation entsprechend den geltenden Standards unerlässlich.
- Eine vermehrte, durch das öffentliche Gesundheitssystem refundierte operative Nutzung in der Präventions‑, Reha- und Arbeitsmedizin sowie der Geriatrie sind anzustreben.

## Abkürzungen

Akt: Protein Kinase B
BEM: Betriebliches Eingliederungsmanagement
DIGEST: Deutsche Interdisziplinäre Gesellschaft für Extrakorporale Stoßwellentherapie
ECM: Extrazelluläre Matrix
eNOS: „Endothelial nitric oxide synthase“
ERK: „Extracellular signal-regulated kinase“
ESWT: Extrakorporalen Stoßwellentherapie
HIF-1α: „Hypoxia-inducible factor 1 alpha“
ISMST: International Society for Medical Shockwave Treatment
KI: Künstliche Intelligenz
MAPK: „Mitogen-activated protein kinase“
PI3K: „Phosphoinositide 3‑kinase“
RTW: Return-to-Work
TAZ: „Transcriptional co-activator with PDZ-binding motif“
TRPV4: „Transient receptor potential vanilloid 4“
VEGF: „Vascular endothelial growth factor“
YAP: „Yes-associated protein“
